# Supplementary material for: Neurocognitive Deficits in First-Episode and Chronic Psychotic Disorders: A Systematic Review from 2009 to 2022
Source: Brain Sci. 2023 Feb 10;13(2):299. doi: 10.3390/brainsci13020299 (PMC9954070; doi:10.3390/brainsci13020299)
Supplement: Supplementary file 1 [file brainsci-13-00299-s001.zip › brainsci-2129953-supplementary.pdf]

Supplementary material

Author	Patient Group			Healthy Control Group		
	Characteristics	n (% female)	Age (SD)	characteristics	n (% female)	Age (SD)
Al-Dujaili [1]	CHS-NR<br>CHS-PR	60 (37)<br>55 (27)	36.2 (12.3)<br>36.5 (9.5)	HC	43 (44)	33.2 (11.1)
Al-Hakeim [2]	CHS	120 (40)	41.0 (9.6)	HC	54 (33.3)	37.9 (10.03)
Beck [3]	CHS (Chinese sample)<br>CHS (Danish sample)	35 (54)<br>31 (42)	28.5<br>25.1	HC (Chinese sample)<br>HC (Danish sample)	38 (46)<br>29 (45)	30.3<br>24.4
Bliksted [4]	FES	36 (47)	22.7	HC	36 (47)	22.7
Bosnjak Kuharic [5]	FEP	129 (36)	24	HC	100 (54)	23
Chattopadhyay [6]	CHS	34 (36)	30.9 (8.24)	HC	47 (11)	29.3 (7.91)
Chen [7]	FES	42 (57)	25.2 (6.2)	HC	36 (42)	26.5 (4.4)
Chen [8]	FES<br>CHS	50 (46)<br>158 (51)	22.3 (6.74)<br>28.3 (7.7)	HC	40 (43)	25.3 (6.79)
Correa-Ghisays [9]	CHS	30 (23)	40.8 (10.7)	HC	28 (64)	36.6 (14.5)
Cuesta [10]	FEP	50 (0.3)	25.5 (5.7)	HC<br>S-FEP	24 (0.25)<br>21	23.2 (5.7)<br>24.9 (6.6)
Da Motta [11]	CHS	38 (18)	40.3 (12.51)	HC	97 (54)	35.5 (10.59)
De la Torre [12]	CHS	97 (29)	40 (7.45)	HC	35 (69)	38.6 (17.98)
Eisenacher [13]	HRP	38 (34)	22.9 (4.17)	HC	38 (32)	24.3 (5.78)
Ferretjans [14]	CHS	69 (38)	39.9 (11.8)	HC	43 (16)	41.5 (10.5)
Frommann [15]	HRP-early<br>HRP-late	116 (35)<br>89 (40)	25.6 (6.1)<br>25.3 (6.4)	HC	87 (44)	25.5 (4.4)

Giordano [16]	CHS	114 (29)	36.9 (9.39)	HC	63 (49)	34.4 (12.48)
Guo [17]	FES	51 (35)	22.5 (4.1)	HC	41 (42)	22.8 (3.9)
Hájková [18]	FEP	53 (36)	32 (7.69)	HC	49 (59)	29 (8.64)
He [19]	FEP	115 (54)	25.3 (8.2)	HC	113 (50)	26.6 (8.9)
Konstantakopoulos [20]	CHS	54 (56)	41.6 (8.58)	HC	53 (59)	43.4 (13.14)
Koshiyama [21]	CHS	428 (28)	45.5 (9.5)	HC	283 (51)	44.5 (11.4)
Li [22]	HRP	34 (38)	21.5 (3.5)	HC	37 (51)	20.8 (3.1)
Liu [23]	HRP<br>FES<br>CHS	73 (43)<br>44 (29)<br>34 (50)	23.3 (4.5)<br>23.5 (4.4)<br>27.1 (2.6)	HC	72 (40)	24.0 (2.9)
Liu [24]	FES	31 (45)	25 (6.8)	HC	33 (52)	24.2 (5)
Maes, Sirivichayakul, Kanchanatawan, et al. [25]	CHS-MR<br>CHS-NMR	40 (55)<br>39 (36)	40.9 (10.5)<br>41.1 (11.7)	HC	40 (75)	37.4 (12.8)
Maes, Sirivichayakul, Matsumoto, et al. [26]	CHS-NOS<br>CHS-OS	39 (46)<br>40 (45)	39.5 (11.2)<br>42.5 (10.8)	HC	40 (75)	37.4 (12.8)
Maes [27]	CHS-ND<br>CHS-D	40 (45)<br>40 (48)	41.3 (10.8)<br>40.9 (11.4)	HC	40 (75)	37.4 (12.8)
Mançe ÇaliŞir [28]	CHS	17 (52)	24.6 (3.20)	HC	23 (43)	27.4 (4.09)
McDonald [29]	HRP	101 (76)	22 (4.0)	HC	38 (68)	23.1
Morales-Muñoz [30]	FEP	38 (18)	26.7 (2.39)	HC	38 (61)	30.3 (0.95)
Ngoma [31]	FEP	188 (46)	28.0 (7.1)	HC	153 (57)	28.1 (7.1)
Randers [32]	HRP	50 (56)	23.6 (4.6)	HC	50 (56)	23.5 (4.4)
Saleem [33]	FEP	20 (20)	26.5 (4.3)	HC	15 (20)	23.8 (5.2)

Service [34]	CHS	160 (17)	37.4 (12.8)	HC	717 (63)	36.7 (14.45)
Shi [35]	CHS	230 (51)	38.7 (11.5)	HC	656 (50)	39.3 (11.4)
Tang [36]	CHS-D<br>CHS-ND	51<br>58	50.2 (6.85)<br>47.9 (6.84)	HC	40	46.8 (10.73)
Vignapiano [37]	CHS	145 (32)	36.9 (9.49)	HC	69 (44)	35.9 (12.79)
Wang [38]	FES	81 (42)	30.4 (8.03)	HC	73 (44)	32.5 (10.03)
Wu [39]	FES<br>CHS	79 (46)<br>132 (48)	25.7 (7.8)<br>44.4 (8.7)	HC	124 (48)	44.7 (8.8)
Xiao [40]	FES	58 (47)	25.0 (5.9)	HC	55 (40)	26.5 (6.6)
Xiu [41]	FES<br>CHS	45 (49)<br>35 (49)	24.1 (6.3)<br>44.1 (11.2)	HC	40 (60)	45.1 (12.8)
Yang [42]	FES<br>CHS<br>SZ (= FES + CHS)	21<br>26<br>47 (49)	 <br>30.2 (8.5)	HC	45 (51)	31.8 (8.1)
Yang [43]	FES<br>CHS	34 (44)<br>31 (48)	22.3 (5.21)<br>27.4 (3.71)	HC	35 (54)	22.6 (1.77)
Yang [44]	CHS	32 (50)	30.0 (8.5)	HC	30 (53)	33.5 (9.1)
Zhang [45]	FES	77 (48)	29.2 (9.6)	HC	75 (43)	28.7 (9.1)
Zhang [46]	FES	32 (31)	22.7 (4.0)	HC	29 (41)	22.1 (3.6)
Zhao [47]	FES-R<br>FES-NR	65 (46)<br>45 (42)	29.3 (10.0)<br>31.6 (13.4)	HC	58 (36)	31.4 (12.0)
Zhou, Tang, et al. [48]	CHS-D<br>CHS-ND	33 (0)<br>41 (0)	48.7 (7.68)<br>46 (5.35)	HC	40 (0)	46 (9.47)
Zhou, Yu, et al. [49]	CHS-D<br>CHS-ND	37 (0)<br>38 (0)	49 (7.43)<br>45.9 (5.54)	HC	38 (0)	46.1 (9.11)
Zhou [50]	CHS-D<br>CHS-ND	58 (0)<br>93 (0)	51.5 (9.99)<br>51.3 (8.84)	HC	113 (0)	50.8 (7.77)

Zong [51]	FES	42 (36)	24.9 (4.8)	HC	38 (34)	24.8 (4.56)

Table S1. Study populations characteristics: CHS: chronic schizophrenia, CHS-D: deficit chronic schizophrenia as defined by stable negative symptoms, CHS-ND: non-deficit chronic schizophrenia, CHS-NMR: chronic schizophrenia without motor retardation, CHS-NR: chronic schizophrenia treatment-non-responders, CHS-MR: chronic schizophrenia with motor retardation, CHS-NOS: chronic schizophrenia without oxidative stress, CHS-OS: chronic schizophrenia with oxidative stress, CHS-PR: chronic schizophrenia treatment-partial-responders, HC: heathy controls, HRP: high risk of psychotic disorder, HRP-early: early stages of high risk for psychotic disorder, HRP-late: late stages of high risk of psychotic disorder, FEP: first-episode psychotic disorder, S-FEP: siblings of first-episode patients, FES: first-episode schizophrenia, FES-NR: non-remitted first-episode schizophrenia, FES-R: remitted first-episode schizophrenia, SZ: schizophrenic patients.

References:

1. Al-Dujaili, A.H.; Mousa, R.F.; Al-Hakeim, H.K.; Maes, M. High Mobility Group Protein 1 and Dickkopf-Related Protein 1 in Schizophrenia and Treatment-Resistant Schizophrenia: Associations With Interleukin-6, Symptom Domains, and Neurocognitive Impairments. *Schizophr Bull* **2021**, *47*, 530-541, doi:10.1093/schbul/sbaa136.
2. Al-Hakeim, H.K.; Almulla, A.F.; Maes, M. The Neuroimmune and Neurotoxic Fingerprint of Major Neurocognitive Psychosis or Deficit Schizophrenia: a Supervised Machine Learning Study. *Neurotoxicity research* **2020**, *37*, 753-771, doi:10.1007/s12640-019-00112-z.
3. Beck, K.I.; Simonsen, A.; Wang, H.; Yang, L.; Zhou, Y.; Bliksted, V. Cross-cultural comparison of theory of mind deficits in patients with schizophrenia from China and Denmark: different aspects of ToM show different results. *Nordic journal of psychiatry* **2020**, *74*, 366-373, doi:10.1080/08039488.2020.1723687.
4. Bliksted, V.; Fagerlund, B.; Weed, E.; Frith, C.; Videbech, P. Social cognition and neurocognitive deficits in first-episode schizophrenia. *Schizophr Res* **2014**, *153*, 9-17, doi:10.1016/j.schres.2014.01.010.
5. Bosnjak Kuharic, D.; Makaric, P.; Kekin, I.; Rossini Gajsak, L.; Zivkovic, M.; Ostojic, D.; Silic, A.; Bajic, Z.; Lukacevic Lovrencic, I.; Beezhold, J.; et al. Changes of neurocognitive status in patients with the first-episode psychosis after 18 months of treatment-A prospective cohort study. *Psychiatry research* **2021**, *304*, 114131, doi:10.1016/j.psychres.2021.114131.
6. Chattopadhyay, S.; Patil, N.M.; Nayak, R.B.; Chate, S.S.; Singh, O.P. Neurocognitive Profile in Indian Individuals Genetically at Risk of Schizophrenia. *Indian journal of psychological medicine* **2020**, *42*, 155-161, doi:10.4103/ijpsym.ijpsym_243_19.
7. Chen, S.; Tian, L.; Chen, N.; Xiu, M.H.; Wang, Z.R.; Wang, Y.C.; Tan, Y.L.; Wang, C.Y. More dampened monocytic Toll-like receptor 4 response to lipopolysaccharide and its association with cognitive function in Chinese Han first-episode patients with schizophrenia. *Schizophr Res* **2019**, *206*, 300-306, doi:10.1016/j.schres.2018.11.001.
8. Chen, S.; Liu, Y.; Liu, D.; Zhang, G.; Wu, X. The difference of social cognitive and neurocognitive performance between patients with schizophrenia at different stages and influencing factors. *Schizophrenia Research: Cognition* **2021**, *24*, 100195, doi:10.1016/j.scog.2021.100195.
9. Correa-Ghisays, P.; Sánchez-Ortí, J.V.; Balanzá-Martínez, V.; Selva-Vera, G.; Vila-Francés, J.; Magdalena-Benedito, R.; Victor, V.M.; Escribano-López, I.; Hernández-Mijares, A.; Vivas-Lalinde, J.; et al. Transdiagnostic neurocognitive deficits in patients with type 2 diabetes

mellitus, major depressive disorder, bipolar disorder, and schizophrenia: A 1-year follow-up study. *J Affect Disord* **2022**, *300*, 99-108, doi:10.1016/j.jad.2021.12.074.
10. Cuesta, M.J.; Moreno-Izco, L.; Ribeiro, M.; López-Ilundain, J.M.; Lecumberri, P.; Cabada, T.; Lorente-Omeñaca, R.; Sánchez-Torres, A.M.; Gómez, M.S.; Peralta, V. Motor abnormalities and cognitive impairment in first-episode psychosis patients, their unaffected siblings and healthy controls. *Schizophr Res* **2018**, *200*, 50-55, doi:10.1016/j.schres.2017.10.035.
11. da Motta, C.; Pato, M.T.; Barreto Carvalho, C.; Castilho, P. The neurocognitive and functional profile of schizophrenia in a genetically homogenous European sample. *Psychiatry research* **2021**, *304*, 114140, doi:10.1016/j.psychres.2021.114140.
12. De la Torre, G.G.; Doval, S.; López-Sanz, D.; García-Sedeño, M.; Ramallo, M.A.; Bernal, M.; González-Torre, S. Neurocognitive Impairment in Severe Mental Illness. Comparative study with Spanish Speaking Patients. *Brain sciences* **2021**, *11*, doi:10.3390/brainsci11030389.
13. Eisenacher, S.; Rausch, F.; Ainser, F.; Englisch, S.; Becker, A.; Mier, D.; Fenske, S.; Meyer-Lindenberg, A.; Kirsch, P.; Zink, M. Early cognitive basic symptoms are accompanied by neurocognitive impairment in patients with an 'at-risk mental state' for psychosis. *Early intervention in psychiatry* **2018**, *12*, 586-595, doi:10.1111/eip.12350.
14. Ferretjans, R.; de Souza, R.P.; Panizzutti, B.; Ferrari, P.; Mantovani, L.; de Campos-Carli, S.M.; Santos, R.R.; Guimarães, F.C.; Teixeira, A.L.; Gama, C.S.; et al. Cannabinoid receptor gene polymorphisms and cognitive performance in patients with schizophrenia and controls. *Revista brasileira de psiquiatria (Sao Paulo, Brazil : 1999)* **2022**, *44*, 26-34, doi:10.1590/1516-4446-2020-1650.
15. Frommann, I.; Pukrop, R.; Brinkmeyer, J.; Bechdolf, A.; Ruhrmann, S.; Berning, J.; Decker, P.; Riedel, M.; Möller, H.J.; Wölwer, W.; et al. Neuropsychological profiles in different at-risk states of psychosis: executive control impairment in the early--and additional memory dysfunction in the late--prodromal state. *Schizophr Bull* **2011**, *37*, 861-873, doi:10.1093/schbul/sbp155.
16. Giordano, G.M.; Perrottelli, A.; Mucci, A.; Di Lorenzo, G.; Altamura, M.; Bellomo, A.; Brugnoli, R.; Corrivetti, G.; Girardi, P.; Monteleone, P.; et al. Investigating the Relationships of P3b with Negative Symptoms and Neurocognition in Subjects with Chronic Schizophrenia. *Brain sciences* **2021**, *11*, doi:10.3390/brainsci11121632.
17. Guo, X.; Li, J.; Wang, J.; Fan, X.; Hu, M.; Shen, Y.; Chen, H.; Zhao, J. Hippocampal and orbital inferior frontal gray matter volume abnormalities and cognitive deficit in treatment-naive, first-episode patients with schizophrenia. *Schizophr Res* **2014**, *152*, 339-343, doi:10.1016/j.schres.2013.12.015.
18. Hájková, M.; Knížková, K.; Siroňová, A.; Keřková, B.; Jonáš, J.; Šustová, P.; Dorazilová, A.; Rodriguez, M. Cognitive performance and lifetime cannabis use in patients with first-episode schizophrenia spectrum disorder. *Cogn Neuropsychiatry* **2021**, *26*, 257-272, doi:10.1080/13546805.2021.1924649.
19. He, Z.; Deng, W.; Li, M.; Chen, Z.; Jiang, L.; Wang, Q.; Huang, C.; Collier, D.A.; Gong, Q.; Ma, X.; et al. Aberrant intrinsic brain activity and cognitive deficit in first-episode treatment-naive patients with schizophrenia. *Psychol Med* **2013**, *43*, 769-780, doi:10.1017/s0033291712001638.
20. Konstantakopoulos, G.; Ioannidi, N.; Psarros, C.; Patrikelis, P.; Stefanatou, P.; Kravariti, E. The impact of neurocognition on mentalizing in euthymic bipolar disorder versus schizophrenia. *Cogn Neuropsychiatry* **2020**, *25*, 405-420, doi:10.1080/13546805.2020.1829579.
21. Koshiyama, D.; Miyakoshi, M.; Thomas, M.L.; Joshi, Y.B.; Molina, J.L.; Tanaka-Koshiyama, K.; Sprock, J.; Braff, D.L.; Swerdlow, N.R.; Light, G.A. Unique contributions of sensory discrimination and gamma synchronization deficits to cognitive, clinical, and psychosocial functional impairments in schizophrenia. *Schizophr Res* **2021**, *228*, 280-287, doi:10.1016/j.schres.2020.12.042.
22. Li, R.R.; Lyu, H.L.; Liu, F.; Lian, N.; Wu, R.R.; Zhao, J.P.; Guo, W.B. Altered functional connectivity strength and its correlations with cognitive function in subjects with ultra-high

risk for psychosis at rest. *CNS neuroscience & therapeutics* **2018**, *24*, 1140-1148, doi:10.1111/cns.12865.
23. Liu, Y.; Wang, G.; Jin, H.; Lyu, H.; Liu, Y.; Guo, W.; Shi, C.; Meyers, J.; Wang, J.; Zhao, J.; et al. Cognitive deficits in subjects at risk for psychosis, first-episode and chronic schizophrenia patients. *Psychiatry research* **2019**, *274*, 235-242, doi:10.1016/j.psychres.2019.01.089.
24. Liu, Y.; Bi, T.; Zhang, B.; Kuang, Q.; Li, H.; Zong, K.; Zhao, J.; Ning, Y.; She, S.; Zheng, Y. Face and object visual working memory deficits in first-episode schizophrenia correlate with multiple neurocognitive performances. *Gen Psychiatr* **2021**, *34*, e100338, doi:10.1136/gpsych-2020-100338.
25. Maes, M.; Sirivichayakul, S.; Kanchanatawan, B.; Carvalho, A.F. In schizophrenia, psychomotor retardation is associated with executive and memory impairments, negative and psychotic symptoms, neurotoxic immune products and lower natural IgM to malondialdehyde. *The world journal of biological psychiatry : the official journal of the World Federation of Societies of Biological Psychiatry* **2020**, *21*, 383-401, doi:10.1080/15622975.2019.1701203.
26. Maes, M.; Sirivichayakul, S.; Matsumoto, A.K.; Michelin, A.P.; de Oliveira Semeão, L.; de Lima Pedrão, J.V.; Moreira, E.G.; Barbosa, D.S.; Carvalho, A.F.; Solmi, M.; et al. Lowered Antioxidant Defenses and Increased Oxidative Toxicity Are Hallmarks of Deficit Schizophrenia: a Nomothetic Network Psychiatry Approach. *Molecular neurobiology* **2020**, *57*, 4578-4597, doi:10.1007/s12035-020-02047-5.
27. Maes, M.; Kanchanatawan, B. In (deficit) schizophrenia, a general cognitive decline partly mediates the effects of neuro-immune and neuro-oxidative toxicity on the symptomatome and quality of life. *CNS spectrums* **2021**, 1-10, doi:10.1017/s1092852921000419.
28. Mançe Çalışır, Ö.; Atbaşoğlu, E.C.; Devrimci Özgüven, H.; Ölmez, Ş. Cognitive Features of High-functioning Adults with Autism and Schizophrenia Spectrum Disorders. *Turk psikiyatri dergisi = Turkish journal of psychiatry* **2018**, *29*, 1-10.
29. McDonald, M.; Christoforidou, E.; Van Rijsbergen, N.; Gajwani, R.; Gross, J.; Gumley, A.I.; Lawrie, S.M.; Schwannauer, M.; Schultze-Lutter, F.; Uhlhaas, P.J. Using Online Screening in the General Population to Detect Participants at Clinical High-Risk for Psychosis. *Schizophr Bull* **2019**, *45*, 600-609, doi:10.1093/schbul/sby069.
30. Morales-Muñoz, I.; Jurado-Barba, R.; Fernández-Guinea, S.; Álvarez-Alonso, M.J.; Rodríguez-Jiménez, R.; Jiménez-Arriero, M.A.; Rubio, G. Cognitive impairments in patients with first episode psychosis: The relationship between neurophysiological and neuropsychological assessments. *Journal of clinical neuroscience : official journal of the Neurosurgical Society of Australasia* **2017**, *36*, 80-87, doi:10.1016/j.jocn.2016.10.023.
31. Ngoma, M.; Vansteelandt, K.; Delespaul, P.; Krabbendam, L.; Miezi, S.M.; Peuskens, J. Cognitive deficits in nonaffective functional psychoses: a study in the Democratic Republic of Congo. *Psychiatry research* **2010**, *180*, 86-92, doi:10.1016/j.psychres.2009.10.007.
32. Randers, L.; Jepsen, J.R.M.; Fagerlund, B.; Nordholm, D.; Krakauer, K.; Hjorthøj, C.; Glenthøj, B.; Nordentoft, M. Generalized neurocognitive impairment in individuals at ultra-high risk for psychosis: The possible key role of slowed processing speed. *Brain and Behavior* **2021**, *11*, e01962, doi:10.1002/brb3.1962.
33. Saleem, M.M.; Harte, M.K.; Marshall, K.M.; Scally, A.; Brewin, A.; Neill, J.C. First episode psychosis patients show impaired cognitive function--a study of a South Asian population in the UK. *Journal of psychopharmacology (Oxford, England)* **2013**, *27*, 366-373, doi:10.1177/0269881113477746.
34. Service, S.K.; Vargas Upegui, C.; Castaño Ramírez, M.; Port, A.M.; Moore, T.M.; Munoz Umanes, M.; Agudelo Arango, L.G.; Díaz-Zuluaga, A.M.; Melo Espejo, J.; López, M.C.; et al. Distinct and shared contributions of diagnosis and symptom domains to cognitive performance in severe mental illness in the Paisa population: a case-control study. *The lancet. Psychiatry* **2020**, *7*, 411-419, doi:10.1016/s2215-0366(20)30098-5.

35. Shi, C.; Kang, L.; Yao, S.; Ma, Y.; Li, T.; Liang, Y.; Cheng, Z.; Xu, Y.; Shi, J.; Xu, X.; et al. What is the optimal neuropsychological test battery for schizophrenia in China? *Schizophr Res* **2019**, *208*, 317-323, doi:10.1016/j.schres.2019.01.034.
36. Tang, X.; Zhou, C.; Gao, J.; Duan, W.; Yu, M.; Xiao, W.; Zhang, X.; Dong, H.; Wang, X.; Zhang, X. Serum BDNF and GDNF in Chinese male patients with deficit schizophrenia and their relationships with neurocognitive dysfunction. *BMC psychiatry* **2019**, *19*, 254, doi:10.1186/s12888-019-2231-3.
37. Vignapiano, A.; Koenig, T.; Mucci, A.; Giordano, G.M.; Amodio, A.; Altamura, M.; Bellomo, A.; Brugnoli, R.; Corrivetti, G.; Di Lorenzo, G.; et al. Disorganization and cognitive impairment in schizophrenia: New insights from electrophysiological findings. *Int. J. Psychophysiol.* **2019**, *145*, 99-108, doi:10.1016/j.ijpsycho.2019.03.008.
38. Wang, M.Y.; Ho, N.F.; Sum, M.Y.; Collinson, S.L.; Sim, K. Impact of duration of untreated psychosis and premorbid intelligence on cognitive functioning in patients with first-episode schizophrenia. *Schizophr Res* **2016**, *175*, 97-102, doi:10.1016/j.schres.2016.04.002.
39. Wu, J.Q.; Chen, D.C.; Tan, Y.L.; Xiu, M.H.; De Yang, F.; Soares, J.C.; Zhang, X.Y. Cognitive impairments in first-episode drug-naive and chronic medicated schizophrenia: MATRICS consensus cognitive battery in a Chinese Han population. *Psychiatry research* **2016**, *238*, 196-202, doi:10.1016/j.psychres.2016.02.042.
40. Xiao, W.; Ye, F.; Liu, C.; Tang, X.; Li, J.; Dong, H.; Sha, W.; Zhang, X. Cognitive impairment in first-episode drug-naïve patients with schizophrenia: Relationships with serum concentrations of brain-derived neurotrophic factor and glial cell line-derived neurotrophic factor. *Progress in neuro-psychopharmacology & biological psychiatry* **2017**, *76*, 163-168, doi:10.1016/j.pnpbp.2017.03.013.
41. Xiu, M.-H.; Wang, D.; Chen, S.; Du, X.-D.; Chen, D.-C.; Chen, N.; Wang, Y.-C.; Yin, G.; Zhang, Y.; Tan, Y.-L.; et al. Interleukin-3, symptoms and cognitive deficits in first-episode drug-naïve and chronic medicated schizophrenia. *Psychiatry research* **2018**, *263*, 147-153, doi:10.1016/j.psychres.2018.02.054.
42. Yang, Y.J.; Xiong, J.W.; Zhao, Y.; Zhan, J.Q.; Chen, H.B.; Yan, K.; Hu, M.R.; Yu, B.; Wei, B. Increased plasma asymmetric dimethylarginine is associated with cognitive deficits in patients with schizophrenia. *Psychiatry research* **2016**, *246*, 480-484, doi:10.1016/j.psychres.2016.10.015.
43. Yang, Y.; Liu, Y.; Wang, G.; Hei, G.; Wang, X.; Li, R.; Li, L.; Wu, R.; Zhao, J. Brain-derived neurotrophic factor is associated with cognitive impairments in first-episode and chronic schizophrenia. *Psychiatry research* **2019**, *273*, 528-536, doi:10.1016/j.psychres.2019.01.051.
44. Yang, Y.J.; Luo, T.; Zhao, Y.; Jiang, S.Z.; Xiong, J.W.; Zhan, J.Q.; Yu, B.; Yan, K.; Wei, B. Altered insulin-like growth factor-2 signaling is associated with psychopathology and cognitive deficits in patients with schizophrenia. *PLoS One* **2020**, *15*, e0226688, doi:10.1371/journal.pone.0226688.
45. Zhang, X.Y.; Tang, W.; Xiu, M.H.; Chen, D.C.; Yang, F.D.; Tan, Y.L.; Wang, Z.R.; Zhang, F.; Liu, J.; Liu, L.; et al. Interleukin 18 and cognitive impairment in first episode and drug naïve schizophrenia versus healthy controls. *Brain, behavior, and immunity* **2013**, *32*, 105-111, doi:10.1016/j.bbi.2013.03.001.
46. Zhang, X.; Yao, J.; Lv, Y.; Zhao, X.; Li, Y.; Sui, Y.; Zhiping, D. An Association Study on the Cognitive Function and the Cerebral Grey Matter Volume of Patients with First-Episode Schizophrenia. *Shanghai Arch Psychiatry* **2018**, *30*, 154-167, doi:10.11919/j.issn.1002-0829.217138.
47. Zhao, Y.; Xiao, W.; Chen, K.; Zhan, Q.; Ye, F.; Tang, X.; Zhang, X. Neurocognition and social cognition in remitted first-episode schizophrenia: correlation with VEGF serum levels. *BMC psychiatry* **2019**, *19*, 403, doi:10.1186/s12888-019-2397-8.
48. Zhou, C.; Tang, X.; You, W.; Wang, X.; Zhang, X.; Zhang, X.; Yu, M. Altered Patterns of the Fractional Amplitude of Low-Frequency Fluctuation and Functional Connectivity Between Deficit and Non-Deficit Schizophrenia. *Front Psychiatry* **2019**, *10*, 680, doi:10.3389/fpsyt.2019.00680.

49. Zhou, C.; Yu, M.; Tang, X.; Wang, X.; Zhang, X.; Zhang, X.; Chen, J. Convergent and divergent altered patterns of default mode network in deficit and non-deficit schizophrenia. *Progress in neuro-psychopharmacology & biological psychiatry* **2019**, *89*, 427-434, doi:10.1016/j.pnpbp.2018.10.012.
50. Zhou, C.; Xue, C.; Chen, J.; Amdanee, N.; Tang, X.; Zhang, H.; Zhang, F.; Zhang, X.; Zhang, C. Altered Functional Connectivity of the Nucleus Accumbens Network Between Deficit and Non-deficit Schizophrenia. *Frontiers in Psychiatry* **2021**, *12*, doi:10.3389/fpsyt.2021.704631.
51. Zong, X.; Zhang, Q.; He, C.; Huang, X.; Zhang, J.; Wang, G.; Lv, L.; Sang, D.; Zou, X.; Chen, H.; et al. DNA Methylation Basis in the Effect of White Matter Integrity Deficits on Cognitive Impairments and Psychopathological Symptoms in Drug-Naive First-Episode Schizophrenia. *Front Psychiatry* **2021**, *12*, 777407, doi:10.3389/fpsyt.2021.777407.
